# Ultrasteep Slope Cryogenic FETs Based on Bilayer Graphene

**DOI:** 10.1021/acs.nanolett.4c02463

**Published:** 2024-09-04

**Authors:** Eike Icking, David Emmerich, Kenji Watanabe, Takashi Taniguchi, Bernd Beschoten, Max C. Lemme, Joachim Knoch, Christoph Stampfer

**Affiliations:** † JARA-FIT and 2nd Institute of Physics, RWTH Aachen University, 52074 Aachen, Germany; ‡ Research Center for Electronic and Optical Materials, National Institute for Materials Science, 1-1 Namiki, Tsukuba 305-0044, Japan; § Research Center for Materials Nanoarchitectonics, National Institute for Materials Science, 1-1 Namiki, Tsukuba 305-0044, Japan; ⊥ Chair of Electronic Devices, RWTH Aachen University, 52074 Aachen, Germany; ∥ IHT, RWTH Aachen University, 52074 Aachen, Germany; || Peter Grünberg Institute (PGI-9), Forschungszentrum Jülich, 52425 Jülich, Germany; # AMO GmbH, 52074 Aachen, Germany

**Keywords:** Bernal stacked bilayer graphene, band gap, subthreshold slope, disorder

## Abstract

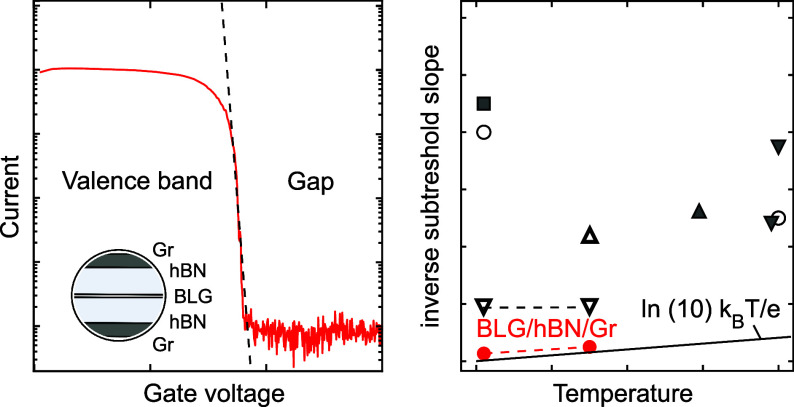

Cryogenic field-effect transistors (FETs) offer great potential for applications, the most notable example being classical control electronics for quantum information processors. For the latter, on-chip FETs with low power consumption are crucial. This requires operating voltages in the millivolt range, which are only achievable in devices with ultrasteep subthreshold slopes. However, in conventional cryogenic metal-oxide-semiconductor (MOS)FETs based on bulk material, the experimentally achieved inverse subthreshold slopes saturate around a few mV/dec due to disorder and charged defects at the MOS interface. FETs based on two-dimensional materials offer a promising alternative. Here, we show that FETs based on Bernal stacked bilayer graphene encapsulated in hexagonal boron nitride and graphite gates exhibit inverse subthreshold slopes of down to 250 μV/dec at 0.1 K, approaching the Boltzmann limit. This result indicates an effective suppression of band tailing in van der Waals heterostructures without bulk interfaces, leading to superior device performance at cryogenic temperature.

Field-effect transistors operable at cryogenic temperatures are an ongoing area of research with potential applications in outer space electronic devices,^1−5^ semiconductor-superconducting coupled systems,^6^ scientific instruments such as infrared sensors,^5,7,8^ and notably control electronics in quantum computing.^9−14^ The distinct advantages of operating at cryogenic temperatures include reduced power dissipation, minimized thermal noise, and faster signal transmission.^1,15,16^ The significance of cryogenic control electronics is especially apparent in the context of quantum information processing, where the availability of control electronics in close proximity to the qubits is seen as a necessary condition for operating large quantum processors with thousands of qubits.^11,14,17−20^ However, developing cryogenic electronics for quantum computing applications poses significant challenges due to the limited cooling power of dilution refrigerators. One of the requirements is to reduce the operational voltage range of the FETs into the mV range,^21^ which, in turn, requires devices with ultrasteep subthreshold slopes. Temperature broadening effects impose a lower limit–the so-called Boltzmann limit–to the inverse subthreshold slope (SS) given by SS_BL_ = *k*_B_*T*/*e* · ln(10), where *T* is the operating temperature and *k*_B_ the Boltzmann constant. Thus, the inverse SS is expected to decrease from 60 mV/dec at room temperature to as low as, e.g., 20 μV/dec at 0.1 K. However, experiments with conventional FET devices optimized for low-temperature operation have shown that the inverse SS saturates at considerably higher values in the order of 10 mV/dec at cryogenic temperature.^22−25^ This saturation originates mainly from static disorder at the metal-oxide-semiconductor (MOS) interface (due to, e.g., surface roughness, charged defects,etc.).^23,24,24−27^ This contributes to the formation of a finite density of states (DOS) near the band edges, which decays exponentially into the band gap.^28^ This so-called band-tailing leads to deteriorated off-state behavior and limits the achievable SS. This effect is further enhanced by dopants, which could either freeze out or become partially ionized.^21,29^ Interface engineering can improve the MOS interface,^30^ but in MOSFETs based on bulk materials, inherent disorder at the interfaces and charged defects within bulk dielectrics cannot be fully eliminated.

FETs based entirely on van der Waals (vdW) materials are a promising alternative because these materials offer atomically clean interfaces, as there are no dangling bonds in the vertical direction. Particularly promising for cryogenic applications are vdW-heterostructures based on Bernal stacked bilayer graphene (BLG).^34^ Indeed, it has been shown that by encapsulating BLG into hexagonal boron nitride (hBN) and by placing it on graphite (Gr), it is possible to open a tunable, ultraclean, and spatially homogeneous band gap in BLG by applying an out-of-plane electric displacement field.^31,35,36^ Such BLG-based heterostructures can be seen as an electrostatically tunable semiconductor.^32,37,38^ The high device quality allowed the realization of BLG-based quantum point contacts^38,39^ and quantum dot devices.^40−42^ Further incorporating graphite top gates (tg) instead of state-of-the-art gold top gates in the BLG heterostructures promises a further reduction of disorder as recent publications reported magnetic and even superconducting phases hosted in the valence and conduction bands of BLG.^43−45^ In this work, we demonstrate the enhanced device quality of dual graphite-gated BLG, evident in ultraclean band gaps and ultrasmall inverse subthreshold slopes, establishing vdW-material-based heterostructures as an ideal platform for cryogenic FETs. We use finite bias spectroscopy to show that the band gap tunability is enhanced in pure vdW BLG heterostructures with almost no residual disorder. By extracting the inverse subthreshold slopes, we obtain values as low as 250 μV/dec at *T* = 0.1 K, which is only an order of magnitude larger than the Boltzmann limit of 20 μV/dec at this temperature. These results demonstrate the effective suppression of band tailing, leading to superior cryogenic device behavior of FETs based on vdW materials compared to conventional FETs.

The studied devices are fabricated by a standard dry van-der-Waals transfer technique.^46,47^ The process involves the sequential stacking of hBN, graphite and BLG flakes produced by mechanical exfoliation.^48^ First, a large hBN flake is selected to completely cover the top graphite gate, which is picked up in the second step. The (top) graphite gate is encapsulated in another hBN flake which acts as the top gate dielectric. We then pick up the BLG, a third hBN flake (bottom gate dielectric), and the bottom graphite gate and transfer the vdW heterostructure to a Si^++^/SiO_2_ substrate. The exact thicknesses of the used hBN dielectric layers (mainly ≈20 nm) can be found in the Table S1. Complete encapsulation of the BLG in hBN is essential to prevent degradation and short circuits to the graphite gates. One-dimensional side contacts are then fabricated using electron-beam lithography, CF_4_-based reactive ion etching and metal evaporation followed by lift-off.^46^ A schematic of the final device, including the gating and contacting scheme, is shown in Figure 1a (an optical image can be found in the Figure S1). If not stated otherwise, all measurements were performed at *T* = 0.1 K in a dilution refrigerator with a two-terminal configuration, where we applied the drain-source voltage symmetrically (for more information on the measurement setup, see ref (32)).

**Figure 1:**
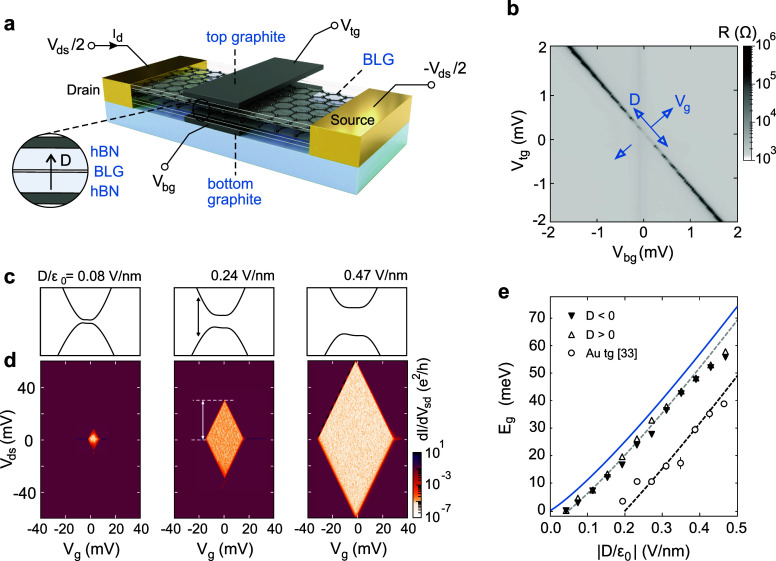
(a) Schematic illustration of a bilayer graphene-based FET. In the active area of the device, the hBN-BLG-hBN heterostructure (see inset) is sandwiched between a top and bottom graphite gate. These gates allow for an independent tuning of the displacement field *D* and the effective gate voltage *V*_g_. The drain-source voltage *V*_ds_ is applied symmetrically in all our measurements. (b) Resistance (*R* = *V*_ds_/*I*_d_) of the BLG as a function of *V*_bg_ and *V*_tg_ at *T* = 1.6 K and *V*_ds_ = 1 mV. The blue arrows indicate the directions of increasing displacement field *D* and *V*_g_. (c) Calculated band structure of BLG around one of the band minima for different displacement fields (see labels). (d) Differential conductance d*I*/d*V*_ds_ as a function of *V*_ds_ and *V*_g_ (at *T* = 0.1 K) for different displacement fields (see labels in c). The band gap *E*_g_ can be extracted from the extension of the diamond along the *V*_ds_ axis (see label). (e) Extracted *E*_g_ as a function of |*D*/ε_0_|. The experimental data are in good agreement with theory calculated according to ref (31) using ε_BLG_ = 1 (blue line) including an offset of 5 meV (gray dashed line). Note that for the same displacement field, the achieved band gap is almost 20 meV higher compared to state-of-the-art BLG devices with gold top gates (open circles taken from ref (32)).

As a first electrical characterization, we measure the drain current *I*_d_ as a function of top and bottom gate voltage by applying a small drain-source voltage *V*_ds_ = 100 μV. Figure 1b shows the resulting map of the BLG resistance R = *V*_ds_/*I*_d_. Here, we observe a diagonal feature of increased resistance with a slope β = 1.22, which gives us directly the relative gate lever arm β = α_bg_/α_tg_, where α_bg_ and α_tg_ denote the gate lever-arms of the top and bottom gate and can be extracted from quantum Hall measurements^49−51^ (for more information, see Supporting Information). The increasing width of the region of maximum resistance with increasing gate voltages is direct evidence for the formation and tuning of the BLG band gap with increasing out-of-plane displacement field *D* (see also band structure calculations in Figure 1c). The displacement field in the dual-gated BLG-based vdW heterostructure is given by *D* = *eα*_tg_[β(*V*_bg_ – *V*_bg_^0^) – (*V*_tg_ – *V*_tg_^0^)]/2, and the effective gate voltage is given by *V*_g_ = [β(*V*_*bg*_ – *V*_bg_^0^) + (*V*_tg_ – *V*_tg_^0^)]/(1 + β), which tunes the electrochemical potential in the band gap of the BLG, μ ≈ *eV*_g_.^32^ Here, ε_0_ is the vacuum permittivity, and the parameters *V*_tg_^0^ and *V*_bg_^0^ account for the offsets of the charge neutrality point from *V*_tg_ = *V*_bg_ = 0.

To study the band gap opening in our devices as a function of the displacement field *D*, we perform finite bias spectroscopy measurements and investigate the differential conductance *dI*/*dV*_ds_ as a function of the effective gating potential *V*_g_ and the applied drain-source voltage *V*_ds_ for different fixed displacement fields *D*, see Figure 1d. A distinct diamond-shaped region of suppressed conductance emerges, which has a high degree of symmetry and sharp edges and scales well with the applied displacement field. The outlines of the diamonds (black dashed lines in Figure 1d) show a slope of ≈2, highlighting that *V*_g_ directly tunes the electrochemical potential μ within the band gap and indicating that the band gap is as good as free of any trap states.^32^ In the Supporting Information. we show that the slope of the diamond outlines is indeed constant (≈ 2) for all displacement fields *D*/ε_0_ ≳ 0.2 V/nm.

From the extension of the diamonds on the *V*_ds_ axis, we can directly extract the size of the band gap *E*_g_,^32^ which are shown in Figure 1e for positive (filled triangles) and negative displacement fields (empty triangles). They agree reasonably well with the theoretical prediction assuming an effective dielectric constant of BLG of ε_BLG_ = 1 (blue line, for more information, see Supporting Information.) except for a small offset of 5 meV (gray dashed line), which might be due to some residual disorder or interaction effects. Measurements on a second graphite top-gated device reveal the same behavior (see Figure S9).

In Figure 1e we also report the results of measurements performed on a similar BLG device but with the top gate made of gold instead of graphite (see ref (32)). It is noteworthy that the extracted band gap for the device with graphite gates is almost 20 meV higher than that extracted for the device with a gold top gate for the same displacement fields, highlighting the importance of clean vdW-interfaces. Furthermore, the observed extracted band gap *E*_g_ persists down to lower displacement fields *D*/ε_0_ ≈ 50 mV/nm compared to devices with a gold top gate.

The high tuning efficiency of the band gap in graphite dual-gated BLG combined with the high symmetry of the diamonds from the bias spectroscopy measurements demonstrates that BLG heterostructures built entirely from vdW materials, including top and bottom gates, outperform BLG devices with non-vdW materials thanks to much cleaner interfaces, allowing them to achieve unprecedented levels of device quality.

The finite bias spectroscopy measurements show that the edges of the diamonds are sharply defined, which promises excellent switching efficiency of FETs based on dual graphite-gated BLG when using *V*_g_ as the tuning parameter. To extract the inverse subthreshold slope, we measure the drain current *I*_d_ as a function of *V*_g_ for fixed *D*-field and *V*_ds_ ≈ 0.1 mV at both band edges, see Figures 2a and 2b. From the linear fits of the slopes (black dashed lines), we extract the inverse subthreshold slope SS = (∂(log_10_(*I*_d_)/∂*V*_g_)^−1^. The resulting values for the valence and conduction band are plotted in Figure 2c.

**Figure 2:**
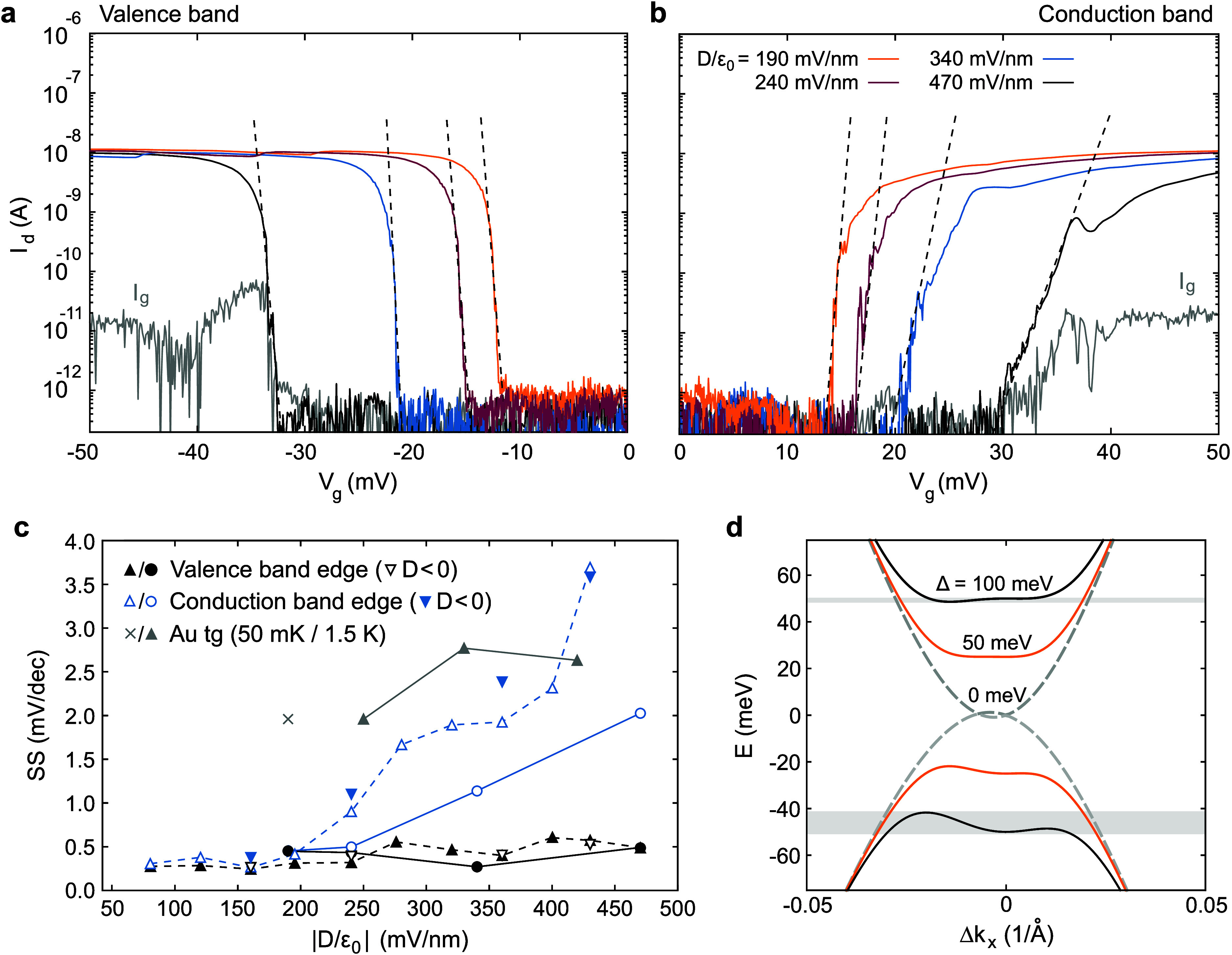
(a, b) Drain current as a function of *V*_g_ for four different displacement fields (see different colors and labels in panel b) near the valence band edge (panel a) and the conduction band edge (panel b). The gate leakage current is shown as the gray trace exemplarily for *D*/ε_0_ = 470 mV/nm (see also Figure S4). Measurements were taken at *V*_ds_ = 0.1 mV and *T* = 0.1 K. (c) Extracted minimal inverse subthreshold slope as a function of the displacement field for both, the valence (black) and conduction (blue) band edges. The black-filled circles and blue circles correspond to data directly extracted from the measurements shown in panels a and b (see black dashed lines), respectively. The upward-pointing triangles are extracted from similar measurements at slightly higher *V*_ds_ ≈ 0.5 mV. Both measurements result in values around 0.3 mV/dec at the valence band edge. At the conduction band edge the S*S*_min_ values show an increase with increasing *D*. Downward-pointing triangles denote SS extracted for negative displacement fields. The gray symbols represent the SS extracted from two devices with a gold top gate at the valence band edge (cross: first device measured at 50 mK, gray upward-pointing triangles: second device measured at 1.5 K). (d) Calculated band structure for different onsite potential differences Δ between the BLG layers. Δ*k*_*x*_ represents the momentum relative to the K and K’ points. Due to trigonal warping effects,^33^ the bands show an asymmetric deformation if a band gap is present. With increasing onsite potential difference, the asymmetry of the deformation increases, indicating a possible origin of the asymmetry in inverse subthreshold slope values.

At the valence band edge, we extract record low values of SS ≈ 270–500 μV/dec, roughly 1 order of magnitude above the Boltzmann limit SS_BL_(0.1 K) = 20 μV/dec. For comparison, the saturation limit of conventional FETs based on non-vdW materials at *T* ≈ 0.1 K is in the order of a few mV/dec.^25^ We repeat similar measurements for slightly higher drain-source voltages *V*_ds_ ≈ 0.5 mV. The results are also shown in Figure 2c as upward-pointing triangles. They agree overall with the values from the measurements at *V*_ds_ = 0.1 mV, with inverse subthreshold slopes at the valence band around SS ≈ 250 to 500 μV/dec. The very low SS value indicates that band tailing is suppressed for devices with only vdW interfaces. This is also supported by the fact that samples with a gold top gate (i.e., an interface between a vdW and a bulk material) show significantly higher SS values for comparable *D*-fields at the valence band edge (see the cross and gray upward-pointing triangles in Figure 2c).

It is remarkable to observe that while the SS extracted at the valence band edge does not show a significant dependency on the applied displacement field *D*, the values extracted at the conduction band edge show a considerable increase from SS ≈ 500 μV/dec up to SS ≈ 2.8 mV/dec with increasing *D*. This displacement field-dependent asymmetry of the SS values is related to the electron–hole asymmetry of the BLG band structure. In principle, this asymmetry could also be due to a top-bottom asymmetry of (weak) interface disorder in the vdW heterostructure, since transport near the band edges is dominated by orbitals in only one of the two graphene layers. For example, for a positive *D*-field, transport at the conductance (valence) band edge is carried only by the top (bottom) layer of the BLG.^32^ Changing the *D*-field direction reverses the band-edge to layer assignment. This allows us to experimentally exclude such a possible nonuniformity of the interface disorder, as we observe the same asymmetry in the SS values for the conductance and valence band edge also for negative *D*-fields (see downward pointing triangles in Figure 2c), in good agreement with the values for positive *D*-fields, thus strongly emphasizing the importance of the asymmetry in the BLG band structure. In Figure 2d we show the calculated band structure as a function of the onsite potential difference between the layers Δ(*D*), which can be directly tuned with the applied displacement field *D* (for more information on the calculations, see Supporting Information). With increasing Δ(*D*), the bands undergo an increasingly asymmetric deformation due to the trigonal-warping effect.^33,52^ As a consequence, the bands change from a hyperbolic shape at low Δ(*D*) to an asymmetric Mexican-hat shape for high Δ(*D*),^31^ see Figure 2d. With increasing band deformation, parts of the bands close to the *K* and *K*′ points of the Brillouin zone become flat. Recent studies have shown that these flat bands give rise to a rich phase diagram in BLG, where magnetic and superconducting phases emerge.^43−45^ The emerging phases could act phenomenologically similar to the interface-induced disorder, resulting in effective tail states at the band edges and degradation of the SS. The flat parts of the bands are right at the conduction band edge, but slightly deeper in the valence band: for example, for Δ = 100 meV in Figure 2d, the local valence band maximum is much more pronounced than the local conduction band minimum (see gray shaded areas). Consequently, the resulting phase diagrams also exhibit an asymmetry similar to our SS values,^45^ which suggests that the asymmetric band deformation could cause the SS asymmetry in our measurements. We observed the same behavior for a second device, although at slightly different *D*-fields (see Figure S10), most likely due to sample-to-sample variations. Regardless, we would like to emphasize that this consistent asymmetry is in itself an indicator of the overall low disorder in our devices.

While our device presents excellent SS values, the measured on–off ratio in Figure 2a and b is only about 10^4^ to 10^5^, which is a direct consequence of the low on-current of about *I*_d_ ≈ 10^–8^ A. This low current level is partially due to the small size of the device contacts, which are circularly etched vias through the hBN, with a diameter of just 1 μm. However, it is mainly because the measured current is limited by our measurement setup, which is optimized for low-noise, small-current measurements but also imposes a sharp limit of about 10^–8^ A, see Figure 3a. In a different setup at higher temperatures *T* = 1.5 K, we observe on-currents of up to 1 μA in the very same device for large *V*_ds_ = 30 mV, see Figure 3b, indicating that higher currents are possible also with the contact geometry used. This is also confirmed by measurements in a second device of similar design, where we measure currents up to 1 μA even at *T* = 0.1 K in a different low-temperature setup (see Figure S11).

**Figure 3:**
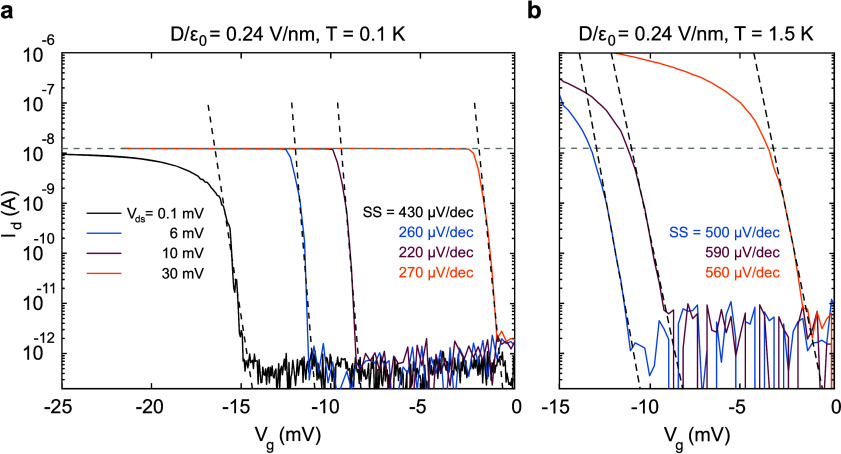
(a, b) Drain current as a function of *V*_g_ at the valence band edge for different applied drain-source voltages *V*_ds_ at a fixed displacement field *D*/ε_0_ ≈ 0.24 V/nm. The data shown in panel a were taken in a dilution refrigerator at *T* = 0.1 K, while those presented in panel b were taken in a pumped ^4^He cryostat at *T* = 1.5 K. The first setup limits the on-current to roughly 10^–8^ A. The second system allows higher on-currents of 1 μA. However, we observe a higher noise level resulting in a slightly increased off-current.

The measurements presented in Figure 3 also show that the threshold voltage shifts to lower values of *V*_g_ with increasing *V*_ds_, without significantly affecting SS, see Figure 3a (more data are provided in Sec. 3 in the Supporting Information). This implies that–despite the small on-current–the device presented in this manuscript could be operated at *T* = 0.1 K as a FET with an on–off ratio of at least 10^5^ and an operational voltage range of only 3–4 mV by suitably choosing the drain-source voltage *V*_ds_, thanks to the small SS ≈ 250 μV/dec At *T* = 1.5 K, reaching an on–off ratio of 10^5^ will require operational voltages of 6–7 mV due to a slightly higher noise level and slightly higher SS ≈ 500 μV/dec.

Finally, we summarize in Figure 4 the minimum inverse SS for different transistor device architectures reported in the literature (empty dots) as a function of temperature for low *T* ≤ 6 K. The best performing conventional FET devices, based on silicon-on-insulator^18^ or nanowires,^53^ allow to reach SS ≈ 2 mV/dec. These values are almost an order of magnitude higher than the 250 μV/dec of the BLG-based devices reported in this work (red dots). The theoretical Boltzmann limit is included as a solid line. At *T* = 1.5 K, the Boltzmann limit is SS_BL_ ≈ 300 μV/dec, only slightly less than the inverse subthreshold slope of our device (SS ≈ 500 μV/dec). We attribute this improvement in SS directly to the reduced interface disorder in devices based on pure vdW heterostructures, i.e., without bulk interfaces to metal or oxides. The detrimental effect of bulk interfaces is well illustrated by the much higher SS values of BLG devices, where the top gate was made of gold instead of graphite (blue triangles in Figure 4). A BLG device with an additional Al_2_O_3_ between the metal top gate and the top hBN performed even worse (green triangle).

**Figure 4:**
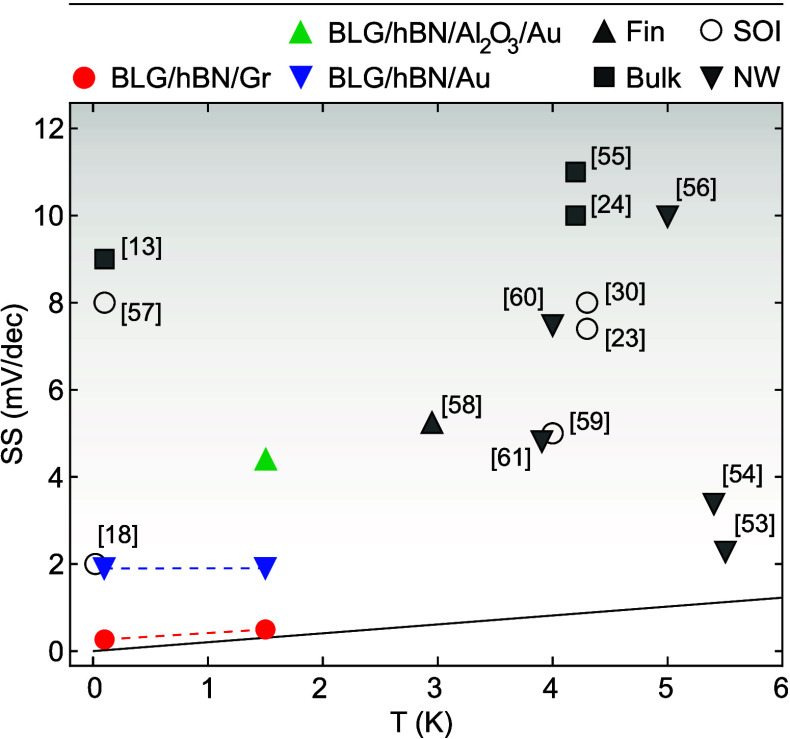
Comparison of the extracted low-temperature SS values for different types of FET devices. The red dots correspond to the device presented in this paper. The blue triangles refer to a similar device but where the top gate was made of gold instead of graphite, and the green triangle refers to a third BLG device with an additional Al_2_O_3_ layer between the hBN and the gold gate. The empty symboles correspond to SS values reported in the literature for FETs based on different technologies (silicon on insulator (SOI), bulk CMOS, Fin, and nanowire FETs^13,18,23,25,30,53−61^). FETs based on vdW heterostructures outperform all other technologies in terms of SS at cryogenic temperatures. The solid black line is the theoretical Boltzmann limit SS_BL_ = *k*_B_*T*/*e* ln(10).

In summary, we have demonstrated that BLG devices based on pure vdW materials exhibit excellent band gap tunability and have provided evidence that 2D material-based FETs offer superior device behavior at cryogenic temperatures, with SS in the order of 250 μV/dec, only 1 order of magnitude above the Boltzmann limit of SS_BL_ ≈ 20 μV/dec at *T* = 0.1 K. The ability to also electrostatically confine carriers in BLG^38,40,42^ and the excellent performance as a field-effect transistor make this type of device an ideal platform for cryogenic applications and calls for further device design improvements that allow for down-scaling and circuit integration. Moreover, we expect this work to trigger the exploration of pure vdW heterostructure FETs based on true 2D semiconductors, such as the transition metal dichalcogenides MoS_2_ and WSe_2_.

## Data Availability

The data supporting the findings are available in a Zenodo repository under accession code 10.5281/zenodo.10526847.

## Supporting Information Available

Equations used to calculate the band gap and band structure in bilayer graphene as a function of the displacement field, additional information for the first sample, comparable data for a second device, and the drain-current traces for the BLG devices with Au top gate and additional Al_2_O_3_ dielectric (PDF)
