# Supplementary material for: Ultrasteep Slope Cryogenic FETs Based on Bilayer Graphene
Source: Nano Lett. 2024 Sep 4;24(37):11454–61. doi: 10.1021/acs.nanolett.4c02463 (PMC11421093; doi:10.1021/acs.nanolett.4c02463)
Supplement: Supplementary file 1 — nl4c02463_si_001.pdf [file nl4c02463_si_001.pdf]

Supporting Information: Ultra-steep slope cryogenic FETs based on bilayer graphene

Eike Icking,[†,⊥] David Emmerich,[†,⊥] Kenji Watanabe,[‡] Takashi Taniguchi,[¶] Bernd Beschoten,[†] Max C. Lemme,[§,#] Joachim Knoch,[∥] and Christoph Stampfer[*,†,⊥]

†*JARA-FIT and 2nd Institute of Physics, RWTH Aachen University, 52074 Aachen, Germany, EU*

‡*Research Center for Electronic and Optical Materials, National Institute for Materials Science, 1-1 Namiki, Tsukuba 305-0044, Japan*

¶ *Research Center for Materials Nanoarchitectonics, National Institute for Materials Science, 1-1 Namiki, Tsukuba 305-0044, Japan*

§*Chair of Electronic Devices, RWTH Aachen University, 52074 Aachen, Germany, EU*

∥*IHT, RWTH Aachen University, 52074 Aachen, Germany, EU*

⊥*Peter Grünberg Institute (PGI-9), Forschungszentrum Jülich, 52425 Jülich, Germany, EU*

#*AMO GmbH, 52074 Aachen, Germany, EU*

* E-mail: stampfer@physik.rwth-aachen.de

In the first section, we present the equations used to calculate the band gap in bilayer graphene as a function of the displacement field based on a self-consistent approach following Ref.[1] and Ref.[2]. Furthermore, we provide additional information for the first sample

introduced in Figs. 1 and 2 of the main manuscript: in Sec. , we present an optical image of the first device, in Sec. , we present quantum Hall measurements to estimate the gate-lever arms, in Sec. we discuss the slopes of the diamond outlines, in Sec. , we explain how we extract the gate leakage current, and Sec. presents some drain current traces for higher temperatures. As mentioned in the main text, additional information regarding the calculated band structure shown in Fig. 2d can be found in Sec. . In Sec. , we present comparable data for a second device, similar to what we have shown in the main text for the first device. Finally, in Sec. , we show the drain-current traces for the BLG devices with Au top gate and additional Al_2O_3 dielectric.

Band gap as a function of displacement field

The band gap in BLG[1,3]

$$E_{\mathrm{g}}(\Delta) = \frac{|\Delta|}{\sqrt{1+(\Delta/\gamma_1)^2}}, \tag{1}$$

with the interlayer coupling strength $\gamma_1 \approx 0.38\,\mathrm{eV}$ and the onsite potential difference[4–7]

$$\Delta = \frac{d_0 e D}{\varepsilon_0 \varepsilon_{\mathrm{BLG}}} + \frac{d_0 e^2}{2\varepsilon_0 \varepsilon_{\mathrm{BLG}}} \delta n(\Delta), \tag{2}$$

depends on the strength of the applied displacement field D. Here, $d_0 = 0.34\,\mathrm{nm}$ is the interlayer spacing of BLG, and ε_0 is the vacuum permittivity[1]. The effective dielectric constant of BLG $\varepsilon_{\mathrm{BLG}}$ accounts for the electric susceptibility of each layer and the corresponding dielectric polarization[8].

Optical Image of the first device

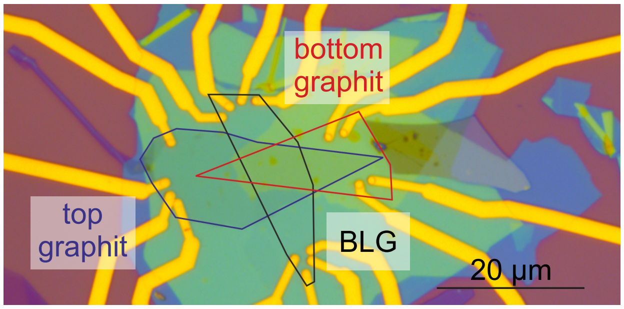

Figure S1: Optical image of the first device discussed in the main text. The BLG and the graphite gates are highlighted. The dual gated area is roughly $6 \times 9\,\mu\text{m}^2$.

Quantum Hall measurements and gate lever-arm

The gate-lever arm

$$\alpha_{\text{tg,bg}} = \frac{\nu e B}{h V_{\text{tg,bg}}}, \tag{3}$$

can be extracted from quantum hall measurements by fitting the Landau level (see Fig.)[9–12]. Here, ν is the Landau filling factor, B the applied magnetic field, h the Planck constant, and $V_{\text{tg(bg)}}$ the applied gate voltage. A full list of the gate-lever arms, the relative gate lever arm (extracted from resistance maps as in Fig. 1c), and the thicknesses of the dielectrics can be found in Table S1 for both devices with graphite top and bottom gates.

Table S1: List of bottom gate lever arm extracted from QH measurements as depicted in Fig. S2, relative lever arm from resistance maps (Fig. 1c), and hBN thicknesses from AFM measurements for both devices with a graphite top and bottom gate.

Sample	$\alpha_{\text{bg}}(10^{11}\,\text{V}^{-1}\text{cm}^{-2})$	β	$\text{d}_{\text{hBN}}^{\text{top}}$ (nm)	$\text{d}_{\text{hBN}}^{\text{bottom}}$ (nm)
1 (main text)	8.4	1.22	17	22
2 (Supp. Mat.)	8.5	0.62	32	19

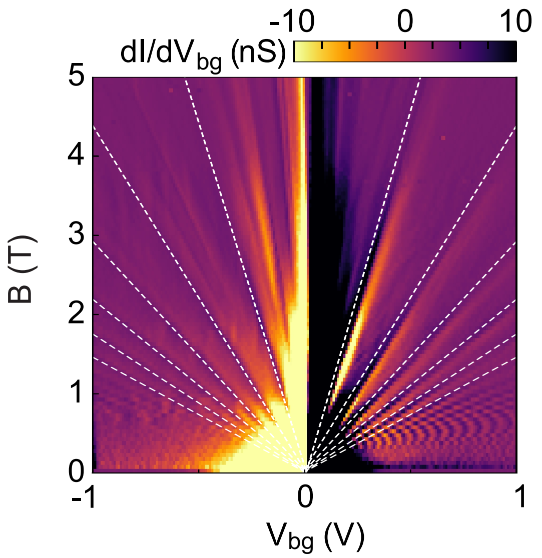

Figure S2: Transconductance as a function of out-of-plane magnetic field and bottom gate voltage. Fitting the Landau level (white dashed line) allows to extract the gate-lever arm.

Slopes of diamond outlines

The slope of the outline of the diamonds of suppressed conductance obtained from the finite bias spectroscopy measurement should have, in the ideal case, a slope of 2[13]. A reduction in slope indicates that it is no longer possible to have a direct tuning of the electrochemical potential μ in the band gap, i.e., $\Delta V_{\mathrm{g}} > \Delta\mu/e$. Interestingly, when plotting the extracted slope of the outline of the diamond (extracted by a fixed resistance threshold value $10^9\,\Omega$, exactly as also used in Ref.[13]) as a function of the displacement field D, we observe a constant slope of very close to 2 for $|D/\varepsilon_0| \gtrsim 0.2\,\mathrm{V/nm}$, see Fig. S3. For smaller displacement fields ($D/\varepsilon_0 < 0.2\,\mathrm{V/nm}$) our method of extracting the slope of the diamond outline breaks down, resulting in smaller values (as shown in Fig. S3). This breakdown is mainly due to the rigidly fixed resistance threshold combined with the rather poor measurement resolution (see e.g. the leftmost panel in Fig. 1d in the main manuscript). In short, for such small displacement fields, the resolution of the diamonds is unfortunately insufficient to fully resolve any sharp structures that would allow reliable extraction of the slope of the diamond outline. As a result, the edge appears smeared, resulting in a decreasing slope value.

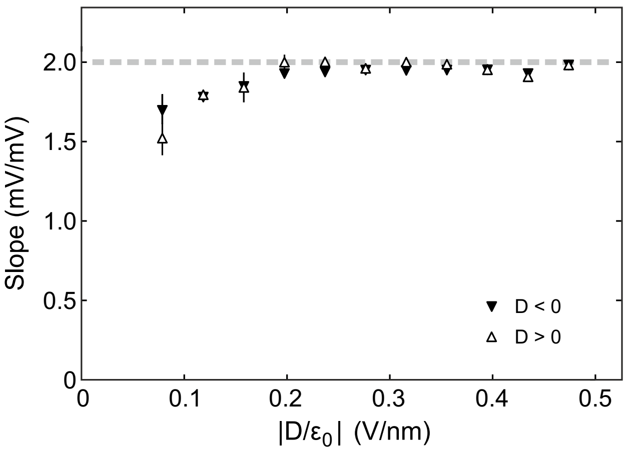

Figure S3: The slopes of the outline of the diamonds (see black dashed lines in Fig. 1d in the main manuscript) obtained from finite bias spectroscopy measurements as a function of the applied displacement field $|D/\varepsilon_0|$. For details on the slope-extraction method, see Ref. [13].

Extracting the gate current

In the experiments, we apply the drain-source voltage symmetrically using an IV converter, which allows us to measure the source current I_s and the drain current I_d simultaneously. We can estimate the gate current $I_\mathrm{g} \approx \Delta I$ from the difference $\Delta I = |I_\mathrm{d} - I_\mathrm{s}|$ between these two currents. Fig. S4 shows the comparison between I_d and I_g for the four displacement fields, as shown in Fig. 2 in the main text.

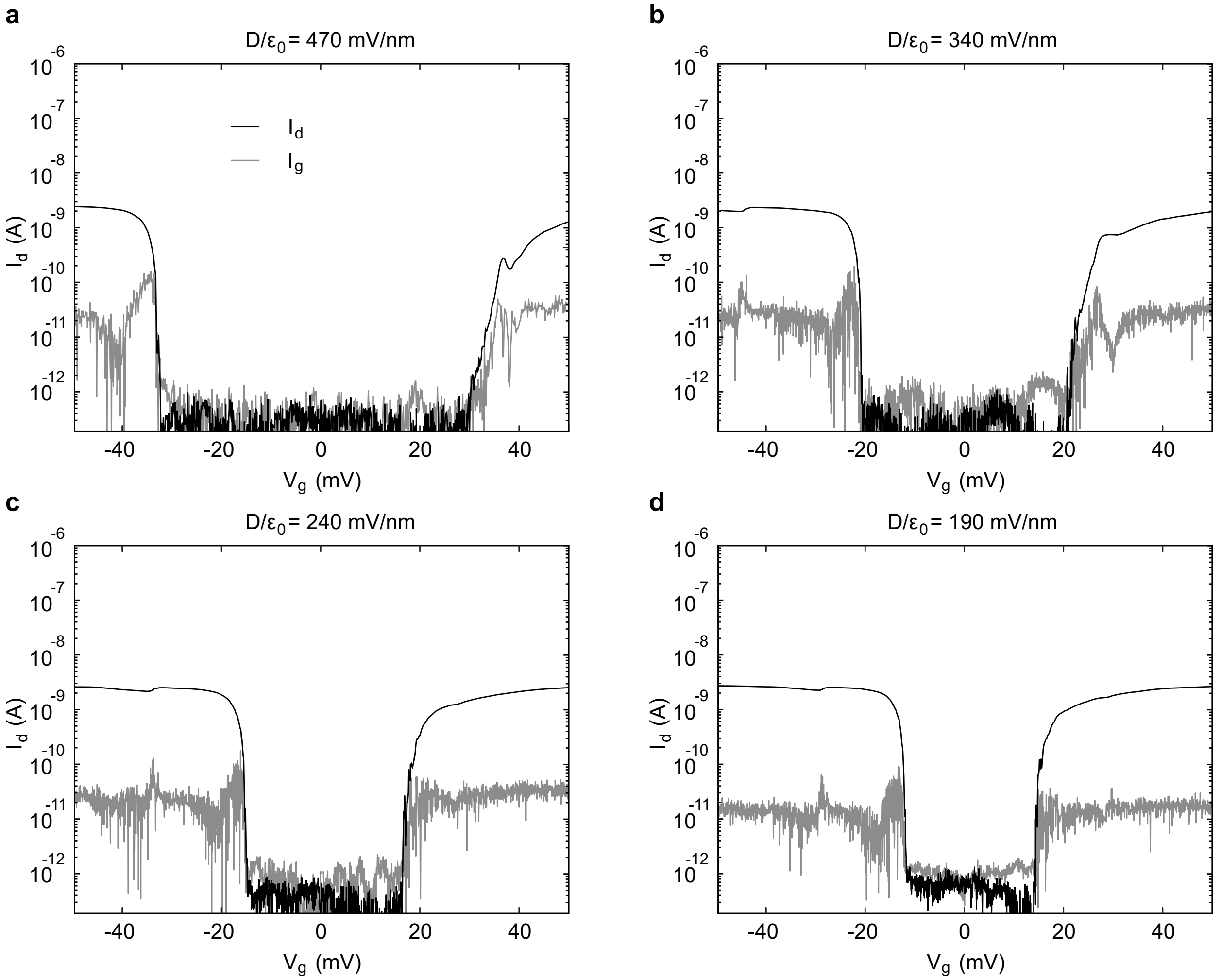

Figure S4: Drain-current (black) and gate current (gray) as a function of effective gating voltage V_{g} at a temperature of $T = 0.1\,\mathrm{K}$ at $V_{\mathrm{ds}} = 0.1\,\mathrm{mV}$ in the cases of the four different displacement fields as depicted in Fig. 2 in the main text.

Subthreshold slope at 1.5 K

In Fig. 4, data points are depicted obtained at $T = 1.5\,\mathrm{K}$ for the first device in a pumped ^{4}He cryostat (see main text). In Fig. S5, we show full line traces for two different displacement fields. Even at $T = 1.5\,\mathrm{K}$, we still see a slight asymmetry in the extracted subthreshold slopes at conduction and valence band edge.

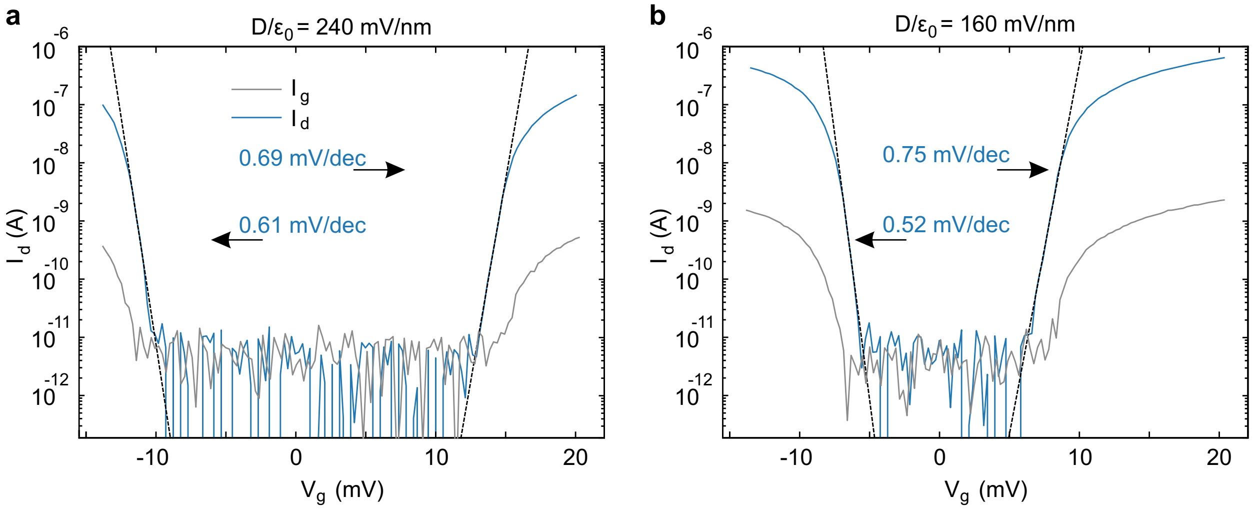

Figure S5: **a,b** Drain-current as a function of effective gating voltage V_g at a temperature of $T = 1.5\,\mathrm{K}$ obtained in a VTI cryostat at $V_\mathrm{ds} = 6\,\mathrm{mV}$ at $D/\varepsilon_0 = 0.24\,\mathrm{V/nm}$ (panel a) and $0.16\,\mathrm{V/nm}$ (panel b). From the fit (black dashed line), the subthreshold slope for the two displacement fields can be extracted for the valence and conduction band edge (indicated by black arrows). The gate current for the same displacement fields is shown in gray.

Subthreshold Slope as a function of V_ds

Fig. 3 in the main text depicts the drain-current for a fixed displacement field $D/\varepsilon_0 = 0.24\,\mathrm{V/nm}$ as a function of V_g for three different V_ds. In order to verify that the SS is not affected by V_ds we performed a more detailed analysis, see Fig. S6. The first sample shows SS $\approx$ 200-350 μV/dec without any dependence on V_ds.

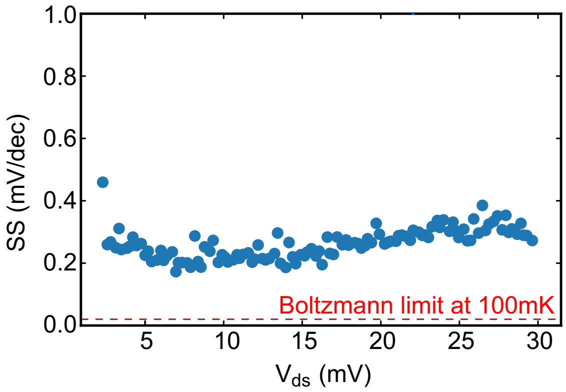

Figure S6: The extracted subthreshold slope for sample 1 for $D/\varepsilon_0 = 0.27\,\mathrm{V/nm}$ as a function of applied drain-source voltage V_ds show an almost constant value of 250 μV/dec.

Bandstructure Calculations

The bandstructure is calculated numerically using the Hamiltonian[14]

$$H = \begin{pmatrix} \Delta/2 & v_0\pi^\dagger & -v_4\pi^\dagger & -v_3\pi \\ v_0\pi & \Delta/2+\Delta' & \gamma_1 & -v_4\pi^\dagger \\ -v_4\pi & \gamma_1 & -\Delta/2+\Delta' & v_0\pi^\dagger \\ -v_3\pi^\dagger & -v_4\pi & v_0\pi & -\Delta/2 \end{pmatrix}, \tag{4}$$

with $\pi \equiv \hbar(\xi q_x + iq_y)$ and $\xi = \pm 1$. The parameter $v_i \equiv \frac{\sqrt{3}a}{2\hbar}\gamma_i$ is defined for each coupling parameter γ_i. These include the intralayer coupling $\gamma_0 = 3.16$ eV, the intralayer coupling $\gamma_1 = 0.381$ eV between dimer sites on different layers, $\gamma_3 = 0.38$ eV accounts for coupling between non-dimer atoms on the two different layers, $\gamma_4 = 0.14$ eV for coupling between non-dimer atoms with dimer site atoms on the other layer, $\Delta' = 0.015$ meV is the energy difference between dimer and non-dimer sites, and Δ the onsite-potential difference, responsible for the band gap formation[6,14].

Second device

Here, we introduce a second device (see an optical image in Fig. S7a). This section shows the analog measurement for the measurements shown in Fig 1 and 2. From the resistance measurements (Fig. S7b), we extract the relative gate lever-arm (see Tab. S1), from the quantum hall measurements (Fig. S7c), we extract the bottom gate lever-arm (see Tab. S1). The finite bias spectroscopy (see Fig. S8) also exhibits clean and highly symmetric diamonds. The extracted band gaps (see Fig. S9) are in excellent agreement with the results from the main text.

We further measure the drain current for different displacement fields, similar to the analysis performed in Fig. 2 in the main text for the first device (see Fig. S10a-e). The extracted subthreshold slopes (see Fig. S10f) also show an asymmetry for values extracted at the

valence and the conduction band edge, which increases with increasing displacement field. This effect is even more pronounced for the second device as we already have a significant difference of roughly 1 mV/dec at $D/\varepsilon_0 \approx 150\,\mathrm{mV/nm}$. The first device shows a comparable difference at slightly higher displacement fields $D/\varepsilon_0 \geq 250\,\mathrm{mV/nm}$. Still, the overall trend is the same for both devices.

Measuring the drain current as a function of V_g for different V_ds exhibits higher on-currents of almost 1 µA, see Fig. S11. In Fig. S12, we show the extended analysis of SS as a function of V_ds for the second device, which yields SS values between 250 µV/dec up to 1 mV/dec, without any significant dependency on V_ds. However, the spread in SS is slightly higher compared to the first device.

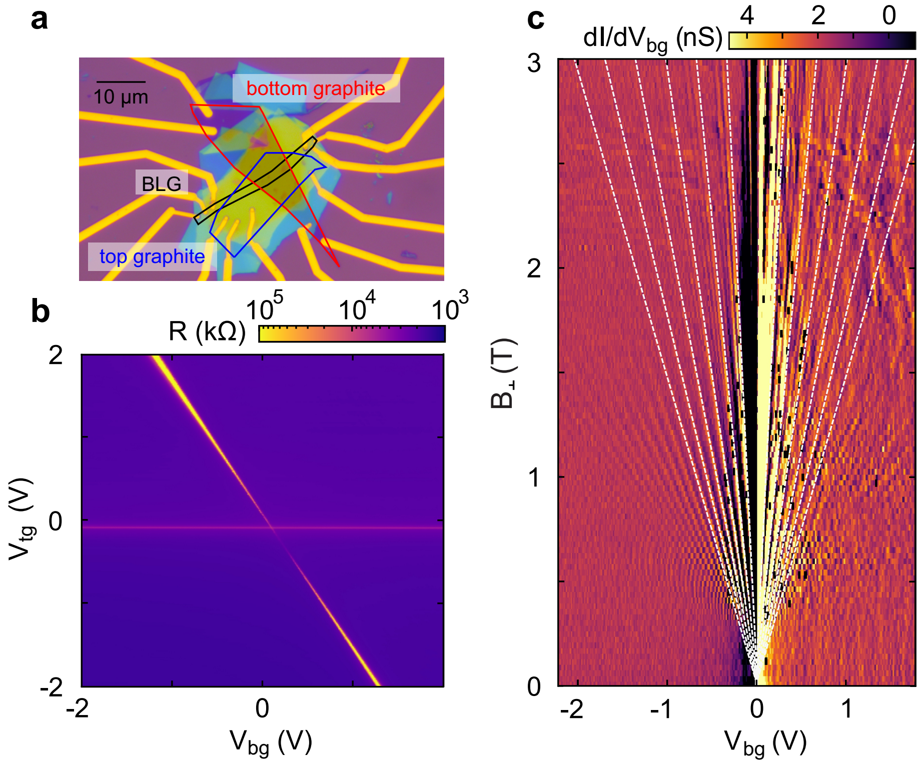

Figure S7: **a** Optical image of a second device. The BLG and the graphite flakes are highlighted. **b** Resistance as a function of top and bottom gate voltage. The slope of the diagonal feature of increased resistance is equal to the relative lever-arm β. **c** Transconductance as a function of out-of-plane magnetic field and bottom gate voltage measured for the second device.

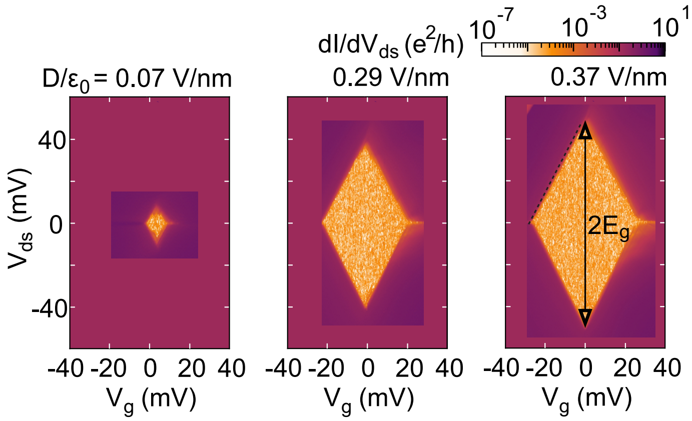

Figure S8: Differential conductance as a function of effective gating voltage V_{g} and drain-source voltage V_{ds} for three different displacement fields measured in a second sample.

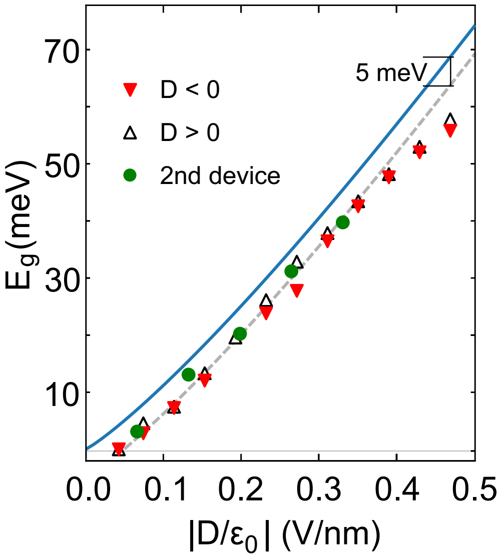

Figure S9: Extracted band gaps from finite bias spectroscopy measurements as a function of the displacement field. The results for the second device (green data points) are in good agreement with the result of the first device (triangles, see also main text). Both data sets follow the theory curve (blue line) if a small offset of about 5 meV is included (dashed line).

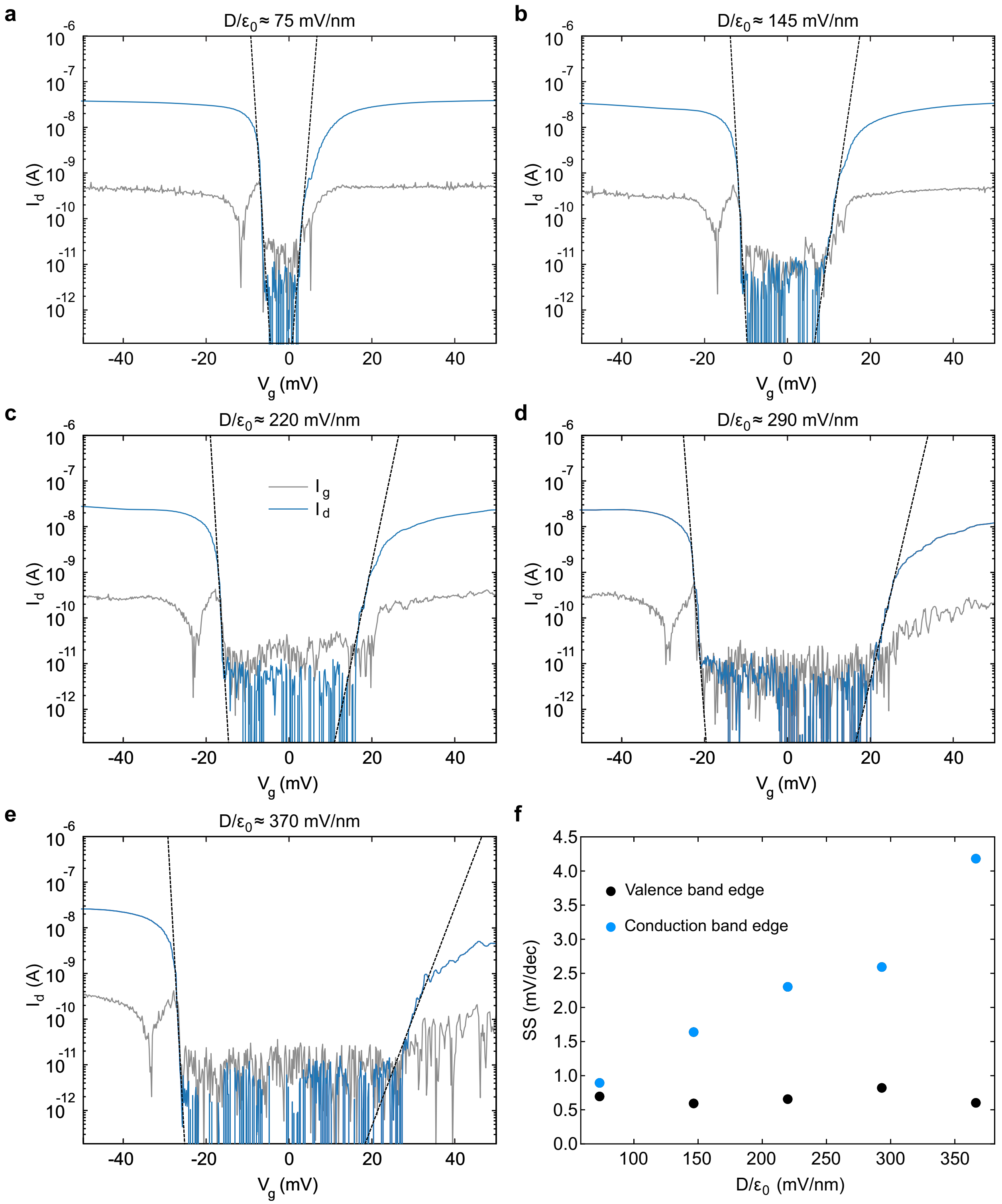

Figure S10: **a** Drain current measured in a second device as a function of effective gating potential V_{g} for five different displacement fields **a** $D/\varepsilon_0 \approx 0.07\,\mathrm{V/nm}$, **b** $0.145\,\mathrm{V/nm}$, **c** $0.22\,\mathrm{V/nm}$, **d** $0.29\,\mathrm{V/nm}$, and **e** $0.37\,\mathrm{V/nm}$. **f** The fits (dashed lines in panels a-e) allow the extraction of the SS as a function of D at the conductance and valence band edge. The same trend as in the main text for device 1 emerges: with increasing D, the SS extracted at the conductance band edge increases, while the SS extracted at the valence band edge stays nearly constant.

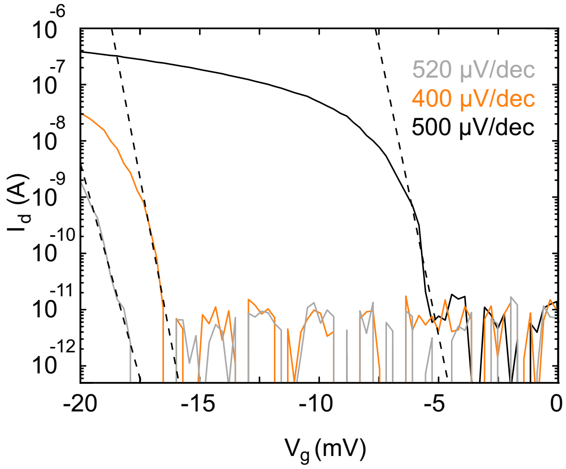

Figure S11: Drain current as a function of effective gating potential V_g at the valence band edge at $T = 0.1\,\mathrm{K}$ measured in a dilution refrigerator for different applied drain-source voltages $V_\mathrm{ds} = 6\,\mathrm{mV}$ (gray), $10\,\mathrm{mV}$ (orange) and $30\,\mathrm{mV}$ (gray) at a fixed displacement field $D/\varepsilon_0 \approx 0.22\,\mathrm{V/nm}$.

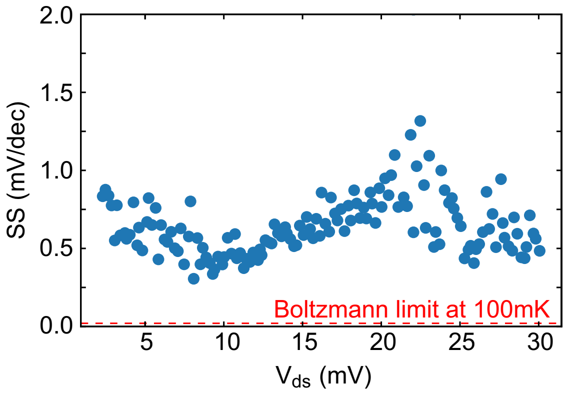

Figure S12: The extracted SS of sample 2 for $D/\varepsilon_0 = 0.27\,\mathrm{V/nm}$ shows a much broader spread than the extracted SS values of sample 1. The extracted values have no clear dependence on the applied V_ds and are more affected by sample-to-sample variations.

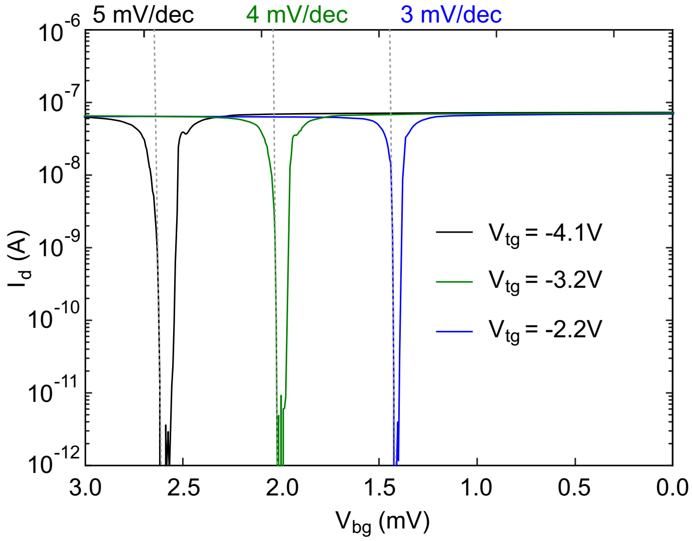

Figure S13: Drain current as a function of bottom gate voltage, while the top gate is fixed to three different voltages. The resulting SS values are considerably worse compared to values extracted from measurements with fixed displacement fields.

Drain current in BLG devices with bulk interfaces

In Fig. 4 of the main text, we present subthreshold slope values for two Gr/hBN/BLG/hBN/Au and one

Gr/hBN/BLG/hBN/Al_2O_3 device. Fig. S14a shows the drain current as a function of effective gate voltage for the first device with an Au top gate measured at $T = 0.1\,\mathrm{K}$, Fig. S14b for the second device with an Au top gate measured at $T = 1.5\,\mathrm{K}$, and Fig. S14c for the Gr/hBN/BLG/hBN/Al_2O_3/Au device measured also at $T = 1.5\,\mathrm{K}$.

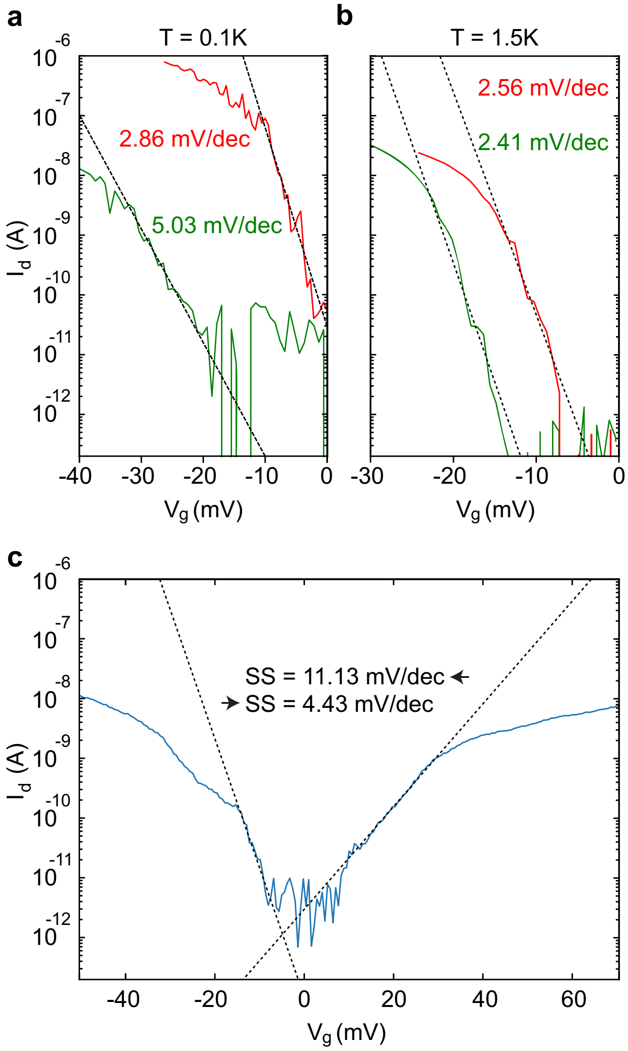

Figure S14: **a** Drain current as a function of effective gating voltage V_g for two displacement fields $D/\varepsilon_0 \approx 0.19\,\mathrm{V/nm}$ (red) and $0.55\,\mathrm{V/nm}$ (green) for a device with an Au top gate, measured at $T = 1.5\,\mathrm{K}$. **b** Drain current for two displacement fields $D/\varepsilon_0 \approx 0.33\,\mathrm{V/nm}$ (red) and $0.42\,\mathrm{V/nm}$ (green) for a second device with an Au top gate, measured at $T = 0.1\,\mathrm{K}$. **c** Drain current measured in a device with an Au top gate and a layer of Al_2O_3 as an extra top gate dielectric (in addition to the top layer of hBN) at $D/\varepsilon_0 \approx 0.6\,\mathrm{V/nm}$ and $T = 1.5\,\mathrm{K}$.

References

(1) McCann, E.; Koshino, M. The electronic properties of bilayer graphene. *Rep. Prog. Phys.* **2013**, *76*, 056503, DOI: 10.1088/0034-4885/76/5/056503.

(2) Slizovskiy, S.; Garcia-Ruiz, A.; Berdyugin, A. I.; Xin, N.; Taniguchi, T.; Watanabe, K.; Geim, A. K.; Drummond, N. D.; Fal'ko, V. I. Out-of-Plane Dielectric Susceptibility of

Graphene in Twistronic and Bernal Bilayers. *Nano Lett.* **2021**, *21*, 6678–6683, DOI: 10.1021/acs.nanolett.1c02211.

(3) Min, H.; Sahu, B.; Banerjee, S. K.; MacDonald, A. H. Ab initio theory of gate induced gaps in graphene bilayers. *Phys. Rev. B* **2007**, *75*, 155115, DOI: 10.1103/PhysRevB.75.155115.

(4) Kuzmenko, A. B.; Benfatto, L.; Cappelluti, E.; Crassee, I.; van der Marel, D.; Blake, P.; Novoselov, K. S.; Geim, A. K. Gate Tunable Infrared Phonon Anomalies in Bilayer Graphene. *Phys. Rev. Lett.* **2009**, *103*, 116804, DOI: 10.1103/PhysRevLett.103.116804.

(5) Joucken, F.; Ge, Z.; Quezada-López, E. A.; Davenport, J. L.; Watanabe, K.; Taniguchi, T.; Velasco, J. Determination of the trigonal warping orientation in Bernal-stacked bilayer graphene via scanning tunneling microscopy. *Phys. Rev. B* **2020**, *101*, 161103, DOI: 10.1103/PhysRevB.101.161103.

(6) Jung, J.; MacDonald, A. H. Accurate tight-binding models for the π bands of bilayer graphene. *Phys. Rev. B* **2014**, *89*, 035405, DOI: 10.1103/PhysRevB.89.035405.

(7) McCann, E. Asymmetry gap in the electronic band structure of bilayer graphene. *Phys. Rev. B* **2006**, *74*, 161403, DOI: 10.1103/PhysRevB.74.161403.

(8) Slizovskiy, S.; Garcia-Ruiz, A.; Drummond, N.; Falko, V. I. Dielectric susceptibility of graphene describing its out-of-plane polarizability. *arXiv* **2019**,

(9) Zhao, Y.; Cadden-Zimansky, P.; Jiang, Z.; Kim, P. Symmetry Breaking in the Zero-Energy Landau Level in Bilayer Graphene. *Phys. Rev. Lett.* **2010**, *104*, 066801, DOI: 10.1103/PhysRevLett.104.066801.

(10) Dauber, J.; Oellers, M.; Venn, F.; Epping, A.; Watanabe, K.; Taniguchi, T.; Hassler, F.; Stampfer, C. Aharonov-Bohm oscillations and magnetic focusing in ballistic graphene rings. *Phys. Rev. B* **2017**, *96*, 205407, DOI: 10.1103/PhysRevB.96.205407.

(11) Sonntag, J.; Reichardt, S.; Wirtz, L.; Beschoten, B.; Katsnelson, M. I.; Libisch, F.; Stampfer, C. Impact of Many-Body Effects on Landau Levels in Graphene. *Phys. Rev. Lett.* **2018**, *120*, 187701, DOI: 10.1103/PhysRevLett.120.187701.

(12) Schmitz, M.; Ouaj, T.; Winter, Z.; Rubi, K.; Watanabe, K.; Taniguchi, T.; Zeitler, U.; Beschoten, B.; Stampfer, C. Fractional quantum Hall effect in CVD-grown graphene. *2D Mater.* **2020**, *7*, 041007, DOI: 10.1088/2053-1583/abae7b.

(13) Icking, E.; Banszerus, L.; Wörtche, F.; Volmer, F.; Schmidt, P.; Steiner, C.; Engels, S.; Hesselmann, J.; Goldsche, M.; Watanabe, K.; Taniguchi, T.; Volk, C.; Beschoten, B.; Stampfer, C. Transport Spectroscopy of Ultraclean Tunable Band Gaps in Bilayer Graphene. *Advanced Electronic Materials* **2022**, *8*, 2200510, DOI: https://doi.org/10.1002/aelm.202200510.

(14) McCann, E.; Koshino, M. The electronic properties of bilayer graphene. *Rep. Prog. Phys.* **2013**, *76*, 056503.
